# Curcumin attenuates proangiogenic and proinflammatory factors in human eutopic endometrial stromal cells through the NF‐κB signaling pathway

**DOI:** 10.1002/jcp.27360

**Published:** 2018-09-27

**Authors:** Indrajit Chowdhury, Saswati Banerjee, Adel Driss, Wei Xu, Sherifeh Mehrabi, Ceana Nezhat, Neil Sidell, Robert N. Taylor, Winston E. Thompson

**Affiliations:** ^1^ Department of Obstetrics and Gynecology Morehouse School of Medicine Atlanta Georgia; ^2^ Department of Physiology Morehouse School of Medicine Atlanta Georgia; ^3^ Nezhat Medical Center Atlanta Center for Minimally Invasive Surgery and Reproductive Medicine Atlanta Georgia; ^4^ Department of Gynecology & Obstetrics Emory University School of Medicine Atlanta Georgia; ^5^ Department of Obstetrics and Gynecology University of Utah School of Medicine Salt Lake City Utah

**Keywords:** curcumin, endometriosis, human, stromal cell

## Abstract

Endometriosis is a chronic gynecological inflammatory disorder in which immune system dysregulation is thought to play a role in its initiation and progression. Due to altered sex steroid receptor concentrations and other signaling defects, eutopic endometriotic tissues have an attenuated response to progesterone. This progesterone‐resistance contributes to lesion survival, proliferation, pain, and infertility. The current agency‐approved hormonal therapies, including synthetic progestins, GnRH agonists, and danazol are often of limited efficacy and counterproductive to fertility and cause systemic side effects due to suppression of endogenous steroid hormone levels. In the current study, we examined the effects of curcumin (CUR, diferuloylmethane), which has long been used as an anti‐inflammatory folk medicine in Asian countries for this condition. The basal levels of proinflammatory and proangiogenic chemokines and cytokines expression were higher in primary cultures of stromal cells derived from eutopic endometrium of endometriosis (EESC) subjects compared with normal endometrial stromal cells (NESC). The treatment of EESC and NESC with CUR significantly and dose‐dependently reduced chemokine and cytokine secretion over the time course. Notably, CUR treatment significantly decreased phosphorylation of the IKKα/β, NF‐κB, STAT3, and JNK signaling pathways under these experimental conditions. Taken together, our findings suggest that CUR has therapeutic potential to abrogate aberrant activation of chemokines and cytokines, and IKKα/β, NF‐κB, STAT3, and JNK signaling pathways to reduce inflammation associated with endometriosis.

## 1 INTRODUCTION

Endometriosis is defined as the growth of endometrial tissue (specifically glands and stroma) outside the uterine cavity, predominantly, but not exclusively, in the peritoneal compartment. It affects an estimated 176 million women, 11% of reproductive age women globally (Adamson, Kennedy, & Hummelshoj, 2010; Buck Louis et al., 2011), and causes mild to severe pelvic pain and infertility (Minici et al., 2008; Vercellini, Viganò, Somigliana, & Fedele, 2014). The pathogenesis of endometriosis is likely multifactorial and several hypotheses have been suggested to explain the presence of ectopic endometrial tissue, such as retrograde menstrual reflux, immune system defects, and the presence of ectopic endometrial stem cells (Burney & Giudice, 2012; Minici et al., 2008; Reis, Petraglia, & Taylor, 2013; Sasson & Taylor, 2008; Vercellini et al., 2014). Extensive investigations have been performed to characterize differences between the eutopic and ectopic endometrium to better understand and define the molecular basis of the disease. To this end, several studies have revealed a distinct pattern of gene expression that regulates cell adhesion, extracellular matrix remodeling, migration, proliferation, immune system regulation, and inflammation in eutopic versus ectopic endometrium (Carli, Metz, Al‐Abed, Naccache, & Akoum, 2009; Carvalho, Podgaec, Bellodi‐Privato, Falcone, & Abrão, 2011; Dai, Leng, Lang, Li, & Zhang, 2012; Eyster, Klinkova, Kennedy, & Hansen, 2007; Honda, Barrueto, Gogusev, Im, & Morin, 2008; Hu, Tay, & Zhao, 2006; Hull et al., 2008; Mihalyi et al., 2010; Wu et al., 2006).

Hormone‐dependent immune system dysregulation through aberrant production of chemokines and cytokines may play a role in endometriosis initiation and progression (Klemmt, Carver, Kennedy, Koninckx, & Mardon, 2006; Meola et al., 2010; Mu et al., 2008; Reis et al., 2013; Ulukus et al., 2009). The symptoms of endometriosis contribute substantially to the burden of disease and add substantial cost to society through expensive surgical and medical therapies and reduced economic productivity (Nnoaham et al., 2011; Simoens et al., 2012; Simoens, Hummelshoj, & Dhooghe, 2007). A definitive diagnosis of endometriosis requires the surgery and the treatment which include pharmacological or operative approaches (Gordts, Puttemans, Gordts, & Brosens, 2015). Unfortunately, current hormonal therapies, including synthetic progestins, GnRH agonists, and danazol are often with limited efficacy and counterproductive to fertility, and they commonly cause systemic side effects (vasomotor symptoms and osteopenia) because of suppression of endogenous steroid hormone levels (Vercellini et al., 2014). Therefore, developing new ways of treating endometriosis using nonhormonal drugs has been a subject of intense investigation. Although many researchers have evaluated individual chemokines and cytokines in patients with endometriosis and matching controls, their exact role in the pathogenesis of the disease remains unsolved (Borrelli, Abrao, & Mechsner, 2014; Borrelli, Hediger et al., 2013; Khorram, Taylor, Ryan, Schall, & Landers, 1993; Li, Luo et al., 2012; Li, Li et al., 2012; Margari et al., 2013). Chemokines represent a family of small cytokines or proteins released by cells, especially lymphocytes, which are capable of inducing chemotaxis (directed movement through chemical gradients in the microenvironment) of nearby responsive cells as a means of directing cellular migration (Burney & Giudice, 2012; Minici et al., 2008; Reis et al., 2013; Vercellini et al., 2014). Inflammatory chemokines are released from a variety of cells in response to bacterial and viral infections and other pathogenic agents that act under both physiological conditions and pathological processes (Acker, Voss, & Timmerman, 1996; Borrelli et al., 2014; Borrelli, Hediger et al., 2013; Lira & Furtado, 2012).

A growing body of experimental evidence suggests that curcumin (CUR) has strong anti‐inflammatory and antioxidant properties (Beevers & Huang, 2011; Lee et al., 2013; Shen & Ji, 2012). CUR (1,7‐bis(4‐hydroxy‐3‐methoxyphenyl)‐1,6‐heptadiene‐3,5‐dione), derived from the rhizomes of Curcuma species plants, is currently undergoing clinical trials for treatment of hormone‐dependent and independent cancers (Beevers & Huang, 2011; Lee et al., 2013; Shen & Ji, 2012). Previously our group demonstrated that the CUR analog, EF24, had strong antiproliferative and antiangiogenic effects on reproductive cells, and did not show any adverse effects on the rat ovarian cycle (Tan et al., 2010). The treatment of human eutopic endometriotic stromal cells (EESCs) with CUR markedly inhibited tumor necrosis factor‐α (TNF‐α)‐induced secretion of interleukin‐6 (IL‐6), IL‐8, monocyte chemotactic protein‐1 (MCP‐1), intercellular adhesion molecule‐1, and vascular cell adhesion molecule‐1, and inhibited the activation of nuclear factor κ‐light‐chain‐enhancer of activated B cells (NF‐κB) transcription factor, a key regulator of inflammation (Kim et al., 2012). In mice, CUR treatment caused a regression of surgically induced ectopic lesions by inhibiting NF‐κB translocation and matrix metalloproteinase expression through accelerated lesion apoptosis, predominantly through the cytochrome *c*‐mediated mitochondrial pathway (Jana, Paul, & Swarnakar, 2012). Interestingly, recent studies have demonstrated that dietary supplements of CUR in combination with standard therapies may lead to the improvement of the regular medical treatment of endometriosis (Signorile, Viceconte, & Baldi, 2018). However, there are no detailed studies of chemokines and cytokines expression profiles in human endometrial stromal cells (ESCs) from normal women and those affected with endometriosis, particularly with respect to the effects of CUR on the secretion of these proteins. Therefore, our current experimental studies were designed to quantify and compare the secretion of chemokine and cytokine from normal endometrial stromal cells (NESC) with that from eutopic endometrium of endometriosis subjects (EESC). We also sought to analyze the ability of CUR to alter chemokines and cytokines secreted from these cells. Immunoblot studies were carried out under various experimental conditions to analyze the phosphorylation and total expression levels of selective inflammatory signaling molecules including inhibitor of nuclear factor κ‐B kinase subunit α/β (IKKα/β), NF‐κB, signal transducer and activator of transcription 3 (STAT3), and c‐Jun N‐terminal kinases (JNK), which are common proinflammatory signaling molecules and upregulated during inflammation (Hoesel, & Schmid, 2013; Huminiecki, Horbańczuk, & Atanasov, 2017; Israël, 2010).

## 2 MATERIALS AND METHODS

### 2.1 Human subjects and tissue acquisition

The current study was approved by the institutional review boards of the Emory and Morehouse Schools of Medicine, Atlanta. Primary ESCs were obtained from reproductive age women with regular menstrual cycles, and who had not received hormonal therapy for at least 3 months before laparoscopic surgery (Yu et al., 2014). Written informed consent was obtained before surgical removal of endometriotic and normal endometrial biopsies. The secretory menstrual phase according to the day of the reproductive cycle was selected for all biopsies to maximize consistency and was confirmed by histological examination of the endometrial tissues. The control and endometriosis subjects were not age‐matched but mean ages were not significantly different between the two groups. For NESC (*n* = 3, controls), endometrial biopsies were obtained from patients undergoing surgery for benign gynecological conditions where there was no visible endometriosis or evidence of endometrial abnormalities confirmed after surgical examination of the abdominal cavity. Among the control subjects, subserosal fibroids were noted and none were greater than 3 cm in diameter. For EESCs (*n* = 3), all patients were found to have surgically identified endometriosis by expert laparoscopists familiar with the varied appearance of the lesions. Histological confirmation of ectopic glands and stroma was confirmed in all endometriosis cases.

### 2.2 ESCs cultures

Primary ESCs from human eutopic endometrial biopsies from three subjects with EESC and three without evidence of endometriosis (NESC) were prepared according to our published procedure (Ryan, Schriock, & Taylor, 1994). All cultures (passages 3–5) were grown in Dulbecco’s modified Eagle’s medium/Ham’s Nutrient Mixture F‐12 (DMEM/Ham’s F‐12; Life Technologies, Inc., Carlsbad, CA) supplemented with 10% fetal bovine serum (FBS; Thermo Fisher Scientific, Grand Island, NY), 1% nonessential amino acids, 1% sodium pyruvate, and 1% penicillin–streptomycin (Sigma‐Aldrich, St. Louis, MO) and incubated at 37°C in a humidified 5% CO_2_ incubator.

### 2.3 CUR treatment of NESC and EESCs

ESC cultures were grown to 95–100% confluence in six‐well plates (Fisher Scientific, Hampton, NH). Cells were treated with CUR (molecular weight = 368.41, purity = 99%; Sigma‐Aldrich) at a concentration of 1, 5, 10, 20, and 40 μg/ml for 24, 48, and 72 hr. CUR was dissolved in dimethyl sulfoxide (DMSO) and diluted to the desired concentrations in DMEM/Ham’s F‐12 media with 5% exosome‐depleted FBS followed by sterilized through 0.22 μm membrane filtration. Exosome‐depleted FBS was obtained by ultracentrifugation of FBS at 100,000*g* for 16 hr at 4°C. The same concentrations of DMSO were added to medium for the parallel vehicle‐control experiments. The final concentration of DMSO was less than 0.1%.

After completion of each experimental group, media were collected and frozen at −80°C for further analysis of chemokine and cytokines as described below.

### 2.4 Assessment of live ESCs after completion of treatments

To assess the morphology of ESCs post‐CUR or vehicle treatment, live cell photographs were taken under an inverted epifluorescence microscope to image the green CUR autofluorescence or the control (untreated) group alone along with phase contrast pictures at ×200 magnification at different times. Following CUR treatment the percentage of survival of both NESC and EESC was determined by nuclear staining with Hoechst 33248 stain as described by Chowdhury et al. (2011).

### 2.5 Assessment of chemokines and cytokines in secretion media

To determine the effects of CUR treatment on NESC and EESC, cytokine and chemokine levels were measured in conditioned media. Culture media were collected at 24 and 48 hr posttreatment of analysis of cytokines (tumor necrosis factor‐α [TNF‐α], vascular permeability factor/vascular endothelial growth factor [VEGF], platelet‐derived growth factor [PDGF], interferon γ [IFNγ], fibroblast growth factors [FGF], interleukin [IL]‐1b (IL‐1β), IL‐1a (IL‐1α), IL‐2, IL‐4, IL‐5, IL‐6, IL‐7, IL‐8, IL‐9, IL‐10, IL‐12, IL‐13, IL‐15, IL‐17) and chemokine (eotaxin [CCL11], granulocyte‐colony stimulating factor [G‐CSF], granulocyte‐macrophage colony stimulating factor [GM‐CSF], IFNγ‐induced protein 10 [IP‐10/CXCL10], MCP‐1/CCL2, macrophage inflammatory proteins 1a [MIP‐1α/CCL3], MIP‐1β/CCL4, RANTES [CCL5]) using Bio‐Plex Pro Human Cytokine, Chemokine, and Growth Factor Magnetic Bead‐Based Assays (BioRad, Hercules, CA) coupled with the Luminex 200 system (Austin, TX) according to the manufacturer’s protocol. Samples were tested at a 1:2 dilution using optimal concentrations of standards and antibodies according to the manufacturer’s protocol.

### 2.6 Western blot (WB) analysis

After various treatments of NESC and EESC, protein were extracted and subjected to one‐dimensional gel electrophoresis and WB analysis (Chowdhury, Branch, Mehrabi, Ford, & Thompson, 2017). For gel electrophoresis, equal amounts of protein (25 mg) were applied to each lane. Primary antibodies were used as described in Table 1. Membranes were incubated with the appropriate secondary antibodies for 2 hr at room temperature and antibody binding was detected by chemiluminescence (Pierce, Rockford, IL). Results of representative chemiluminescence experiments were scanned and densitometrically analyzed using a Power Macintosh Computer (G3; Apple Computer, Cupertino, CA) equipped with a Scan Jet 6100C Scanner (Hewlett‐Packard, Greeley, CO). Quantification of the scanned images was performed using NIH Image version 1.61 software (NIH, Bethesda, MD).

**Table 1:** List of antibodies used for western blot analysis

Peptide/protein target	Name of antibody	Name of individual providing the antibody	Species raised (monoclonal or polyclonal)	Research Resource Identifier (RRID)	Dilution used
Phospho‐nuclear factor κ‐light‐chain‐enhancer of activated B cells (pNF‐κB)	Anti‐phospho‐NF‐kB (pNF‐κB)	Cell Signaling, Beverly, MA	Rabbit monoclonal	AB_331284	1:1,000
Nuclear factor κ‐light‐chain‐enhancer of activated B cells (NF‐κB)	Anti‐NF‐κB (NF‐κB)	Cell Signaling, Beverly, MA	Rabbit monoclonal	AB_10859369	1:1,000
Phospho‐inhibitor of nuclear factor κ‐B kinase subunit β (pIKKβ)	Anti‐phospho‐IKKβ (pIKKβ)	Cell Signaling, Beverly, MA	Rabbit monoclonal	AB_2122301	1:1,000
Inhibitor of nuclear factor κ‐B kinase subunit β (IKKβ)	Anti‐IKKβ (IKKβ)	Cell Signaling, Beverly, MA	Rabbit	AB_11024092	1:1,000
Phospho‐inhibitor of nuclear factor κ‐a kinase subunit α (pIKKα)	Anti‐phospho‐IKKα (pIKKα)	Cell Signaling, Beverly, MA	Rabbit monoclonal	AB_2079382	1:1,000
Inhibitor of nuclear factor κ‐a kinase subunit β (IKKα)	Anti‐IKKα (IKKα)	Cell Signaling, Beverly, MA	Rabbit polyclonal	AB_331626	1:1,000
Phospho‐signal transducer and activator of transcription 3 (pSTAT3)	Anti‐phospho‐STAT3 (pSTAT3)	Cell Signaling, Beverly, MA	Rabbit monoclonal	AB_2491009	1:1,000
Signal transducer and activator of transcription 3 (STAT3)	Anti‐STAT3 (STAT3)	Cell Signaling, Beverly, MA	Rabbit monoclonal	AB_331269	1:1,000
Phospho‐c‐Jun N‐terminal kinase (pJNK)	Anti‐phospho‐JNK (pJNK)	Cell Signaling, Beverly, MA	Mouse monoclonal	AB_2129572	1:1000
c‐Jun N‐terminal kinase (JNK)	Anti‐JNK (JNK)	Cell Signaling, Beverly, MA	Mouse monoclonal	AB_2130165	1:1,000
α Tubulin	Anti‐α tubulin	Sigma‐Aldrich, St. Louis, MO	Mouse monoclonal	AB_477579	1:10,000

### 2.7 Statistical analysis

All experiments were replicated a minimum of three times unless otherwise stated. Data are expressed as mean ± *SEM* of three independent experiments. Statistical analysis was performed by two‐way ANOVA using SPSS version 11.0 software (SPSS, Chicago, IL) to test the significance of differences in CUR dose, duration, and interaction between dose and duration. Post hoc corrections for multiple comparisons were done by Newman–Keuls’ test. Differences were considered significant at *p* ≤ 0.05.

## 3 RESULTS

### 3.1 Intracellular uptake of CUR in normal and EESCs

**Figure 1:**
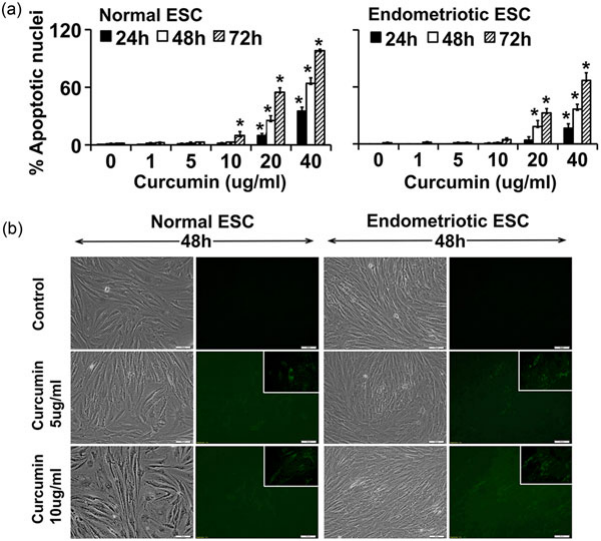
The intracellular uptake of CUR in NESCs and cells derived from EESCs, and their survival status. Cells were cultured and treated with or without CUR (1, 5, 10, 20, and 40 µg/ml) for 24, 48, and 72 hr in DMEM/Ham’s F‐12 media with 5% exosome‐depleted fetal bovine serum. (a) ESCs were fixed and stained with Hoechst 33248 to identify nuclei. Data represent the percentage of cells displaying morphological alteration of apoptosis based on quantification of nuclear morphologic changes. At least 250–300 cells were counted for each data point. The bar graph represents the mean ± *SEM* of results from three independent experiments. Significant (*p* ≤ 0.05) differences are represented with star “*” and compared to the parallel control group. (b) To assess if morphological changes occur in cells, live cell photographs were taken under an inverted epifluorescence microscope to image the green fluorescence signals for the CUR autofluorescence or the control (untreated) group alone along with phase contrast pictures at ×200 magnification at 48 hr posttreatment. Inset images are at a higher magnification, demonstrating CUR autofluorescence. Data are representative of three individual experiments (*n* = 3) from eutopic endometrial biopsies from three subjects with and three without evidence of endometriosis that were performed for each of the two patient groups. CUR: curcumin; DMEM: Dulbecco’s modified Eagle’s medium; EESC: eutopic endometrium of endometriosis subjects; ESCs: endometrial stromal cells; NESCs: normal human endometrial stromal cells [Color figure can be viewed at wileyonlinelibrary.com]

We used our well‐established cell culture model of endometriosis to understand the differential chemokine and cytokine secretory capacity of the cells. Given that the bioavailability of natural CUR is low (Lee et al., 2013; Shen & Ji, 2012), therefore, we first determined the optimum concentration and its intracellular uptake in ESCs (Figure 1). ESCs were grown to 95–100% confluence and treated with different doses (1, 5, 10, 20, and 40 μg/ml) of CUR for 24, 48, and 72 hr. As shown in Figure 1a, the survival of both NESC and EESC cells were evaluated after exposure to different doses of CUR treatment of different time points. The effect of CUR was potent and significant on ESCs. CUR caused apoptotic cell death in a dose‐dependent and time‐dependent manner (*p* < 0.05; Newman–Keuls’ test). Indeed, there was a 100% apoptotic cell death at 72 hr in response to 40 μg/ml of CUR (*p* < 0.05). However, lower doses (<20 μg/ml) of CUR had no significant apoptotic effects on ESCs. These results further suggest that EESCs are significantly more resistant to cell death compare to NESCs (Dmowski, Gebel, & Braun, 1998). Thus, based on these results we selected 5 and 10 μg/ml dose for all other experimental studies.

In addition, we determined cell morphology under various experimental conditions. Phase contrast photomicrograph pictures (Figure 1b) showed that both NESC and EESC have classical mesenchymal characteristics with spindle shaped morphology and oval or round nuclei when grown in exosome free low serum media. As previously reported, under basal conditions there were no significant apparent morphological differences observed between NESC and EESC (Yu et al., 2014). After treatment with CUR for 48 hr, a dose‐dependent increase in green autofluorescence was noted, confirming that CUR was absorbed intracellularly.

### 3.2 Differential secretion of chemokines and cytokines in NESCs versus EESCs

**Figure 2:**
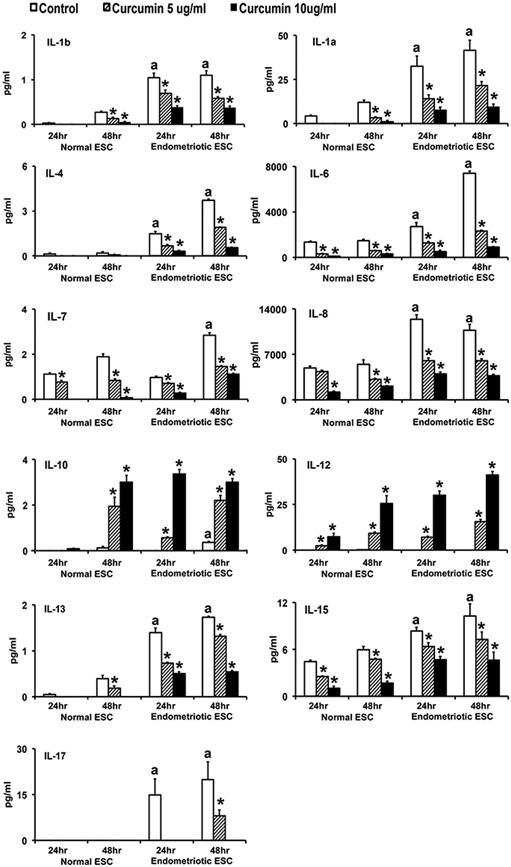
CUR attenuated proinflammatory interleukin secretion from human NESCs and cells derived from EESCs, but not IL‐10 or IL‐12. Cells were treated with or without CUR (5 µg/ml or 10 µg/ml) for 24 and 48 hr in DMEM/Ham’s F‐12 media with 5% exosome‐depleted fetal bovine serum. Basal levels of chemokines and cytokines were measured and analyzed in the supernatants using Bio‐Plex Pro Human Cytokine, Chemokine, and Growth Factor Magnetic Bead‐Based Assays, coupled with the Luminex 200 system. All bar graphs represent the mean ± *SEM* of results from three individual experiments (*n* = 3) from eutopic endometrial biopsies from three subjects with and three without evidence of endometriosis. The superscript “a” represents significant differences (*p* ≤ 0.05) in EESCs groups compared with respective NESCs groups at 24 and 48 hr. Star (*) represents significant differences (*p* ≤ 0.05) in EESCs groups treated with CUR compared with respective NESCs groups treated with CUR at 24 and 48 hr. CUR: curcumin; DMEM: Dulbecco’s modified Eagle’s medium; EESCs: eutopic endometrium of endometriosis subjects; NESCs: normal endometrial stromal cells

**Figure 3:**
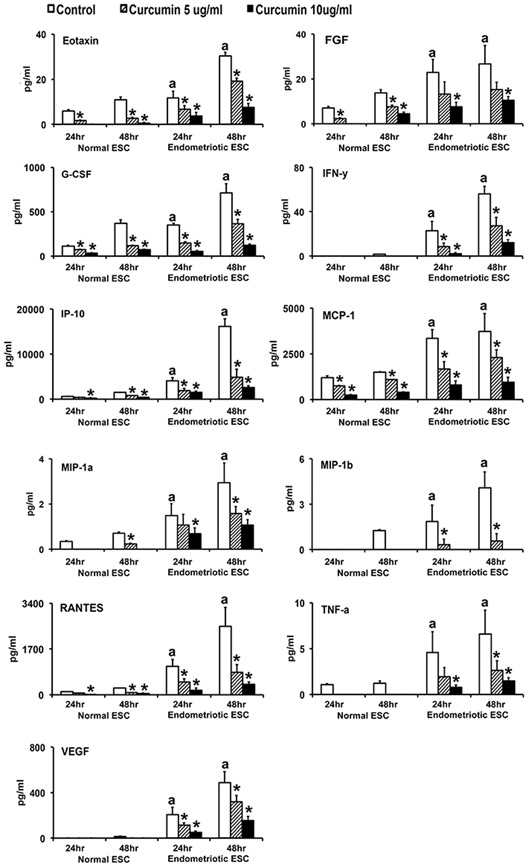
CUR attenuated proinflammatory chemokines and cytokines secreted by human NESCs and cells derived from EESCs. Cells were treated with or without CUR (5 µg/ml or 10 µg/ml) for 24 and 48 hr in DMEM/Ham’s F‐12 media with 5% exosome‐depleted fetal bovine serum. Concentrations of proinflammatory chemokines and cytokines were measured and analyzed in the supernatants using Bio‐Plex Pro Human Cytokine, Chemokine, and Growth Factor Magnetic Bead‐Based Assays, coupled with the Luminex 200 system (R&D System Inc., Minneapolis, MN). All bar graphs represent the mean ± *SEM* of results from three individual experiments (*n* = 3) from eutopic endometrial biopsies from three subjects with and three without evidence of endometriosis. The superscript “a” represents significant differences (*p* ≤ 0.05) in EESCs groups compared with respective NESCs groups at 24 and 48 hr. Star (*) represents significant differences (*p* ≤ 0.05) in EESCs groups treated with CUR compared with respective NESCs groups treated with CUR at 24 and 48 hr. CUR: curcumin; CCL11: chemokine eotaxin; DMEM: Dulbecco’s modified Eagle’s medium; EESC: eutopic endometrium of endometriosis subjects; FGF: fibroblast growth factors; G‐CSF: granulocyte‐colony stimulating factor; GM‐CSF: granulocyte‐macrophage colony stimulating factor; IFNγ: interferon γ; IP‐10/CXCL10: interferon γ‐induced protein 10; MCP‐1/CCL2: monocyte chemotactic protein‐1; MIP‐1a/CCL3: macrophage inflammatory proteins 1a; NESC: normal endometrial stromal cells; PDGF: platelet‐derived growth factor; TNF‐α: tumor necrosis factor‐α; VEGF: vascular permeability factor/vascular endothelial growth factor

As shown in Figures 2, 3 and Table 2, most chemokines and cytokines were secreted in significantly (*p* ≤ 0.05) higher concentrations by EESC compared with NESC at 24 and 48 hr. Some proteins, for example, VEGF, MIP‐1β, and IFNy were at or below the limit of detectability in media from NESC at 24 hr; and IL‐17 was completely absent in media from NESC at 24 and 48 hr. IL‐2, IL‐5, IL‐9, GM‐CSF, and PDGF were not detected in culture media from either EESC or NESC at 24 and 48 hr (not shown). Consistent with previous reports, several chemokines and cytokines were highly overexpressed in EESC (e.g., IL‐6, IL‐8, IP‐10, G‐CSF, MCP‐1, and RANTES were orders of magnitude higher than other chemokine and cytokines in EESC). By contrast, under basal conditions, IL‐10 and IL‐12 expression were not different between EESC and NESC.

**Table 2:** Two‐way ANOVA analysis of CUR dose and duration effects and dose–duration interaction on proinflammatory cytokines and chemokines secretion in human normal endometrial stromal cells (NESCs) and cells derived from eutopic endometrium of endometriosis subjects (EESCs) in vitro

Cytokines and chemokine	Effects of CUR dose		Effects of CUR duration		CUR dose and duration interaction	
IL‐1b (IL‐1β)	*F* (2, 24) = 216.7	*p* < 0.0001	*F* (3, 24) = 301.9	*p* < 0.0001	*F* (6, 24) = 31.93	*p* < 0.0001
IL‐1a (IL‐1α)	*F* (2, 24) = 517.2	*p* < 0.0001	*F* (3, 24) = 308.0	*p* < 0.0001	*F* (6, 24) = 40.62	*p* < 0.0001
IL‐4	*F* (2, 24) = 294.7	*p* < 0.0001	*F* (3, 24) = 487.	*p* < 0.0001	*F* (6, 24) = 111.4	*p* < 0.0001
IL‐6	*F* (2, 24) = 2,123	*p* < 0.0001	*F* (3, 24) = 1,452	*p* < 0.0001	*F* (6, 24) = 456.1	*p* < 0.0001
IL‐7	*F* (2, 24) = 287.7	*p* < 0.0001	*F* (3, 24) = 111.5	*p* < 0.0001	*F* (6, 24) = 14.45	*p* < 0.0001
IL‐8	*F* (2, 24) = 1,904	*p* < 0.0001	*F* (3, 24) = 429.3	*p* < 0.0001	*F* (6, 24) = 109.0	*p* < 0.0001
IL‐10	*F* (2, 24) = 381.1	*p* < 0.0001	*F* (3, 24) = 152.2	*p* < 0.0001	*F* (6, 24) = 57.24	*p* < 0.0001
IL‐12	*F* (2, 24) = 548.2	*p* < 0.0001	*F* (3, 24) = 98.94	*p* < 0.0001	*F* (6, 24) = 43.65	*p* < 0.0001
IL‐13	*F* (2, 24) = 563.5	*p* < 0.0001	*F* (3, 24) = 1,055	*p* < 0.0001	*F* (6, 24) = 109.1	*p* < 0.0001
IL‐15	*F* (2, 24) = 669.0	*p* < 0.0001	*F* (3, 24) = 292.9	*p* < 0.0001	*F* (6, 24) = 11.35	*p* < 0.0001
IL‐17	*F* (2, 24) = 492.9	*p* < 0.0001	*F* (3, 24) = 261.7	*p* < 0.0001	*F* (6, 24) = 170.7	*p* < 0.0001
Eotaxin	*F* (2, 24) = 547.4	*p* < 0.0001	*F* (3, 24) = 537.1	*p* < 0.0001	*F* (6, 24) = 63.56	*p* < 0.0001
FGF	*F* (2, 24) = 52.78	*p* < 0.0001	*F* (3, 24) = 34.67	*p* < 0.0001	*F* (6, 24) = 2.639	*p* = 0.0414
G‐CSF	*F* (2, 24) = 645.0	*p* < 0.0001	*F* (3, 24) = 374.1	*p* < 0.0001	*F* (6, 24) = 88.43	*p* < 0.0001
IFN‐g (IFNγ)	*F* (2, 24) = 579.9	*p* < 0.0001	*F* (3, 24) = 1,163	*p* < 0.0001	*F* (6, 24) = 252.3	*p* < 0.0001
IP‐10	*F* (2, 24) = 1,269	*p* < 0.0001	*F* (3, 24) = 1,435	*p* < 0.0001	*F* (6, 24) = 549.4	*p* < 0.0001
MCP‐1	*F* (2, 24) = 1,829	*p* < 0.0001	*F* (3, 24) = 792.8	*p* < 0.0001	*F* (6, 24) = 95.58	*p* < 0.0001
MIP‐1a (MIP‐1α)	*F* (2, 24) = 1,031	*p* < 0.0001	*F* (3, 24) = 1,489	*p* < 0.0001	*F* (6, 24) = 217.0	*p* < 0.0001
MIP‐1b (MIP‐1β)	*F* (2, 24) = 2,533	*p* < 0.0001	*F* (3, 24) = 1,177	*p* < 0.0001	*F* (6, 24) = 593.3	*p* < 0.0001
RANTES	*F* (2, 24) = 1,167	*p* < 0.0001	*F* (3, 24) = 1,382	*p* < 0.0001	*F* (6, 24) = 454.8	*p* < 0.0001
TNF‐a (TNF‐α)	*F* (2, 24) = 729.4	*p* < 0.0001	*F* (3, 24) = 434.5	*p* < 0.0001	*F* (6, 24) = 38.51	*p* < 0.0001
VEGF	*F* (2, 24) = 215.3	*p* < 0.0001	*F* (3, 24) = 879.8	*p* < 0.0001	*F* (6, 24) = 81.93	*p* < 0.0001

*Note.* CCL11: chemokine eotaxin; FGF: fibroblast growth factors; G‐CSF: granulocyte‐colony stimulating factor; GM‐CSF: granulocyte‐macrophage colony stimulating factor; IFNγ: interferon γ; IL: interleukin; IP‐10/CXCL10: interferon γ‐induced protein 10; MCP‐1/CCL2: monocyte chemotactic protein‐1; MIP‐1α/CCL3: macrophage inflammatory proteins 1α; PDGF: platelet‐derived growth factor; TNF‐α: tumor necrosis factor‐α; VEGF: vascular permeability factor/vascular endothelial growth factor. *F* represents degrees of freedom numerator (Dfn) and degrees of freedom denominator (Dfd) for each group.

### 3.3 CUR treatment attenuates secretion of chemokines and cytokines from NESC and EESC

As shown in Figures 2, 3, and Table 2, CUR treatment inhibited secretion (*p* ≤ 0.05) of nearly all the selected chemokines and cytokines in a concentration and duration dependent manner in both EESC and NESC, except IL‐10 and IL‐12. CUR treatment significantly (*p* ≤ 0.05) inhibited (10–15‐fold) the secretion of IL‐6, IL‐8, IP‐10, G‐CSF, MCP‐1, and RANTES in EESC. By contrast, CUR treatment significantly (*p* ≤ 0.05) promoted the secretion of IL‐10 and IL‐12, particularly from EESC in a dose‐ and time‐dependent manner. Interestingly, higher dose of CUR treatment significantly (*p* ≤ 0.05) promoted the secretion of IL‐10 and IL‐12 in NESC media at 48 hr. The effects of CUR on IL‐17 could not be evaluated in NESC since it was completely absent in media from these cells at both 24 and 48 hr.

### 3.4 CUR treatment attenuates phosphorylation of IKKα, IKKβ, and NF‐κB proteins

**Figure 4:**
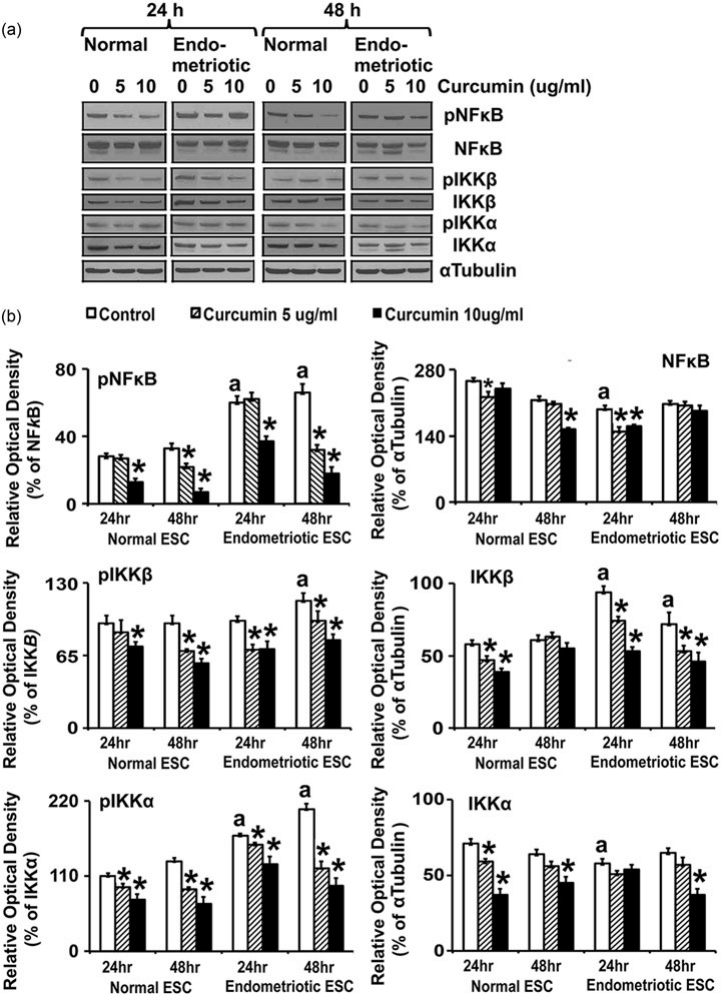
Effects of CUR on phosphorylation and total expression of IKKα, IKKβ, and NF‐κB proteins in human NESCs and cells derived from EESCs subjects. Cells were treated with or without curcumin (CUR, 5 µg/ml or 10 µg/ml) for 24 and 48 hr in DMEM/Ham’s F‐12 media with 5% exosome‐depleted fetal bovine serum. Total protein was isolated, followed by equal amounts of protein (25 μg) from each sample separated by one‐dimensional gel electrophoresis and analyzed for phospho‐IKKα, phospho‐IKKβ, and phospho‐NF‐κB; and total IKKα, IKKβ, and NF‐κB protein. (a) Representative western blot analysis of protein for phospho‐ and total IKKα, IKKβ, and NF‐κB levels in NESCs and EESCs treated with or without CUR. α Tubulin was used as an internal constitutive control. (b) Bar diagrams represent the densitometric analyses of protein in WBs of three independent experiments (*n* = 3) as mean ± *SEM* that were performed for each individual group. The bar graphs represent the ratios of phospho‐IKKα, phospho‐IKKβ, and phospho‐NF‐κB protein levels normalized to total IKKα, IKKβ, and NF‐κB, respectively, and the ratios of total IKKα, IKKβ, and NF‐κB protein levels, normalized to α tubulin. The superscript “a” represents significant differences (*p* ≤ 0.05) in EESCs groups compared with respective NESCs groups at 24 and 48 hr. Star (*) represents significant differences (*p* ≤ 0.05) in EESCs groups treated with CUR compared with respective NESCs groups treated with CUR at 24 and 48 hr. CUR: curcumin; DMEM: Dulbecco’s modified Eagle’s medium; EESCs: eutopic endometriotic stromal cells; IKKα/β: inhibitor of nuclear factor kappa‐B kinase subunit α/β; JNK: c‐Jun N‐terminal kinases; NESCs: normal endometrial stromal cells; NF‐κB: nuclear factor κ‐light‐chain‐enhancer of activated B cells; STAT3: signal transducer and activator of transcription 3; WBs: western blots

The activation of IKKα, IKKβ, and NF‐κB are essential steps for proinflammatory gene expression. Thus, we first evaluated the expression and phosphorylation of IKKα, IKKβ, and NF‐κB in normal and endometriotic ESCs (Figure 4a,b and Table 3). The levels of phosphorylated IKKα and NF‐κB were significantly (*p* ≤ 0.05) higher concentrations in EESCs compared with NESCs at 24 and 48 hr, whereas, phosphorylated IKKβ was significantly higher (*p* ≤ 0.05) concentrations in EESC compared with NESC at 48 hr. Since NF‐κB activity is controlled by the steady‐state levels of IKKα and IKKβ, we analyzed the phosphorylation status of IKKα, IKKβ, and NF‐κB with or without treatment of CUR. Interestingly, CUR treatment inhibited phosphorylation of IKKα, IKKβ, and NF‐κB significantly (*p* ≤ 0.05) in a dose‐ and time‐dependent manner in ESCs. Moreover, higher doses of CUR significantly (*p* ≤ 0.05) inhibited the phosphorylation of IKKα, IKKβ, and NF‐κB in EESCs at 48 hr similar to NESCs.

**Table 3:** Two‐way ANOVA analysis of CUR dose and duration effects and dose–duration interaction on proinflammatory signaling molecules in human normal endometrial stromal cells (NESCs) and cells derived from eutopic endometrium of endometriosis subjects (EESCs) in vitro

Protein name	Effects of CUR dose		Effects of CUR duration		CUR dose and duration interaction	
pNF‐κB/NF‐κB	*F* (2, 24) = 267.5	*p* < 0.0001	*F* (3, 24) = 233.5	*p* < 0.0001	*F* (6, 24) = 21.95	*p* < 0.0001
NF‐κB/tubulin	*F* (2, 24) = 42.42	*p* < 0.0001	*F* (3, 24) = 121.4	*p* < 0.0001	*F* (6, 24) = 21.92	*p* < 0.0001
pIKKβ/IKKβ	*F* (2, 24) = 62.13	*p* < 0.0001	*F* (3, 24) = 19.50	*p* < 0.0001	*F* (6, 24) = 2.244	*p* = 0.0735
IKKβ/tubulin	*F* (2, 24) = 63.17	*p* < 0.0001	*F* (3, 24) = 41.18	*p* < 0.0001	*F* (6, 24) = 7.838	*p* < 0.0001
pIKKα/IKKα	*F* (2, 24) = 209.	*p* < 0.0001	*F* (3, 24) = 138.2	*p* < 0.0001	*F* (6, 24) = 21.56	*p* < 0.0001
IKKα/tubulin	*F* (2, 24) = 76.86	*p* < 0.0001	*F* (3, 24) = 0.7527	*p* = 0.5316	*F* (6, 24) = 8.700	*p* < 0.0001
pSTAT3/STAT3	*F* (2, 24) = 481.5	*p* < 0.0001	*F* (3, 24) = 151.6	*p* < 0.0001	*F* (6, 24) = 32.25	*p* < 0.0001
STAT3/tubulin	*F* (2, 24) = 1.280	*p* = 0.2964	*F* (3, 24) = 29.50	*p* < 0.0001	*F* (6, 24) = 3.072	*p* = 0.0224
pJNK/JNK	*F* (2, 24) = 61.40	*p* < 0.0001	*F* (3, 24) = 37.90	*p* < 0.0001	*F* (6, 24) = 7.230	*p* = 0.0002
JNK/tubulin	*F* (2, 24) = 364.5	*p* < 0.0001	*F* (3, 24) = 1188	*p* < 0.0001	*F* (6, 24) = 126.8	*p* < 0.0001

*Note*. IKKα: inhibitor of nuclear factor κ‐a kinase subunit α; NF‐κB: nuclear factor κ‐light‐chain‐enhancer of activated B cells; pIKKα: phospho‐inhibitor of nuclear factor κ‐a kinase subunit α; pIKKβ: inhibitor of nuclear factor κ‐B kinase subunit β; pJNK: phospho‐c‐Jun N‐terminal kinase; pNF‐κB: phospho‐nuclear factor κ‐light‐chain‐enhancer of activated B cells; pSTAT3: phospho‐signal transducer and activator of transcription 3; STAT3: signal transducer and activator of transcription 3. *F* represents degrees of freedom numerator (Dfn) and degrees of freedom denominator (Dfd) for each group.

### 3.5 CUR treatment attenuates phosphorylation of STAT3 and JNK proteins

**Figure 5:**
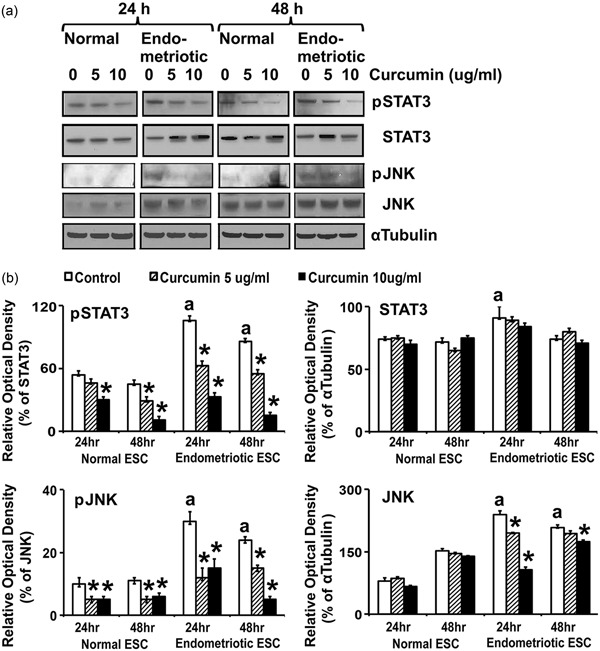
Effects of CUR on phosphorylation and total expression of STAT3 and JNK proteins in human NESCs and cells derived from EESCs subjects. Cells were treated with or without curcumin (CUR, 5 µg/ml or 10 µg/ml) for 24 and 48 hr in DMEM/Ham’s F‐12 media with 5% exosome‐depleted fetal bovine serum. Total protein was isolated, followed by equal amounts of protein (25 μg) from each sample separated by one‐dimensional gel electrophoresis and analyzed for phospho‐STAT3 and phospho‐JNK; and total STAT3 and JNK protein. (a) Representative WBs analysis of protein for phospho‐ and total STAT3 and JNK levels in NESCs and EESCs treated with or without CUR. α Tubulin was used as an internal control. (b) Bar diagrams represent the densitometric analyses of specific protein bands in WBs of three independent experiments (*n* = 3) as mean ± *SEM* that were performed for each individual group. The bar graphs represent the ratios of phospho‐STAT3 and phospho‐JNK protein levels normalized to total STAT3 and JNK, respectively, and the ratios of total STAT3 and JNK protein levels normalized to α tubulin. The superscript “a” represents significant differences (*p* ≤ 0.05) in EESCs groups compared with respective NESCs groups at 24 and 48 hr. Star (*) represents significant differences (*p* ≤ 0.05) in EESCs groups treated with CUR compared with respective NESCs groups treated with CUR at 24 and 48 hr. CUR: curcumin; DMEM: Dulbecco’s modified Eagle’s medium; EESCs: eutopic endometrium of endometriosis; JNK: c‐Jun N‐terminal kinases; NESCs: normal endometrial stromal cells; STAT3: signal transducer and activator of transcription 3; WBs: western blots

The engagement of cell surface cytokine and chemokine receptors activates the JNK, which phosphorylate and activate cytoplasmic STAT proteins (Hoesel & Schmid, 2013; Huminiecki et al., 2017; Israël, 2010). Therefore, we evaluated the expression and phosphorylation of STAT3 and JNK in normal and endometriotic ESCs (Figure 5a,b and Table 3). The levels of phosphorylated STAT3 and JNK were significantly (*p* ≤ 0.05) higher in EESCs compared with NESCs at 24 and 48 hr. Interestingly, CUR treatment significantly inhibited (*p* ≤ 0.05) the phosphorylation of STAT3 and JNK in a dose‐ and time‐dependent manner in EESCs. Moreover, CUR treatment also significantly (*p* ≤ 0.05) decreased the overall expression of JNK (Figure 5a,b and Table 3).

**Figure 6:**
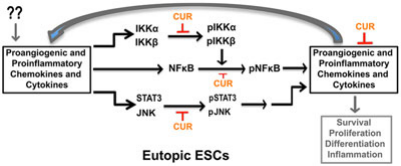
A schematic model showing the antiangiogenic and anti‐inflammatory functions of CUR in human ESCs. Schematic representation of CUR‐dependent inhibition of proangiogenic and proinflammatory chemokine and cytokines may be through inhibited or eliminated phosphorylated forms of JNK and STAT3, along with IKKα, IKKβ, and NF‐κB in human ESCs. CUR: curcumin; ESCs: endometrial stromal cells; IKKα/β: inhibitor of nuclear factor κ‐B kinase subunit α/β; JNK: c‐Jun N‐terminal kinases; NF‐κB: nuclear factor κ‐light‐chain‐enhancer of activated B; STAT3: signal transducer and activator of transcription 3; p—phosphorylated form; arrow represents promotion and blunt arrow represents inhibition [Color figure can be viewed at wileyonlinelibrary.com]

## 4 DISCUSSION

In the current study, we performed a systematic assessment of chemokine and cytokine secretion and confirmed that many of these autacoids are differentially expressed by stromal cells derived from EESC subjects, relative to women without the disease. Interestingly, CUR treatment renders normalization of these proteins, in many cases to the basal secretion levels observed in NESCs. It is well‐established that eutopic endometrial cells function differently in women with endometriosis compared with a normal endometrium in disease‐free women (Burney et al., 2007). These cells are resistant to apoptosis and have other selective advantages for survival outside the uterine cavity, which lead to their implantation and invasion of the peritoneum and other ectopic sites (Dmowski et al., 1998). The detailed identification of molecular differences in the eutopic endometrium of women with endometriosis is an important step toward understanding the pathogenesis of this condition and developing effective strategies for the treatment of its associated infertility and pain. Therefore, we hypothesized that an increase in chemokines, cytokines, and/or, growth factors produced in eutopic endometrial tissue from women with endometriosis may contribute to increases in angiogenesis and proliferation.

Our results indicate that EESCs have an increased basal production of almost all the selected proinflammatory and proangiogenic chemokines and cytokines (except IL‐10) and that they can promote a chronic inflammatory environment within the pelvis of these women (Vercellini et al., 2014). Also, a large body of evidence indicates that TNF‐α, IL‐1β, IFN‐γ, IL‐6, IL‐8, eotaxin, and RANTES are involved in recruitment and activation of macrophages, neutrophils, eosinophils, basophils, monocytes, and NK‐cell to the sites of endometriosis, thus promoting inflammatory changes and enhance angiogenesis through increase production of VEGF (Reis et al., 2013).

Several hormonal treatments and analgesics are available to endometriosis patients suffering pain (Vercellini et al., 2014). The current medical strategies for endometriosis management involve inhibition of ovulation, abolition of menstruation, and achievement of a stable steroid hormone milieu (Vercellini et al., 2014). Creation of hypoestrogenic (GnRH agonists), hyperandrogenic (danazol, gestrinone), or hyperprogestogenic (oral contraceptives, progestins) environments result in the suppression of endometrial and endometriosis cell proliferation. However, serious side effects (vasomotor symptoms, mood instability, and negative calcium balance) and unfavorable changes in serum cholesterol lipoprotein distribution (HDL levels decrease and LDL levels increase) are associated with these therapies. Thus, we chose to evaluate the effects of CUR, a natural, medicinal Asian herb, on proinflammatory and proangiogenic chemokine and cytokine secretion in EESCs and NESCs. Our findings reveal that CUR is a potent inhibitor of proinflammatory and proangiogenic chemokine and cytokine secretion from these cells. By contrast, IL‐10 and IL‐12, which themselves have anti‐inflammatory properties, were upregulated by CUR, particularly in EESCs. Interestingly, the biological actions of these two ILs include inactivation of macrophages and inhibition of proinflammatory and proangiogenic cytokines and chemokines.

Our results further demonstrated that the phosphorylation states of IKKα, IKKβ, NF‐κB, JNK, and STAT3 are higher in EESCs compare with NESCs. The phosphorylation of IKKα and IKKβ involves the successive participation of various kinases linked to cytokine‐specific membrane receptor and chemokine‐specific membrane receptor complexes and adaptor proteins, which converge on NF‐κB signaling pathway (Hoesel, & Schmid, 2013; Huminiecki et al., 2017; Israël, 2010). IKKα and IKKβ are part of a multiprotein complex involved in mediating transcription of multiple chemokine and cytokine genes through I*k*β (Hoesel, & Schmid, 2013; Huminiecki et al., 2017; Israël, 2010). JNK is a member of the mitogen‐activated protein kinase family and cytokine/chemokine‐dependent phosphorylation of JNK modifies the activity of numerous proteins that reside or act in the mitochondria or nucleus. Downstream molecular targets of JNK regulate several important cellular functions including cell growth, differentiation, and survival. Similarly, in response to cytokines, chemokines, and growth factors, STAT3 is phosphorylated by receptor‐associated Janus kinases (JAK), form homodimers or heterodimers, and translocate to the cell nucleus where they act as transcription activators and promote cell proliferation and differentiation (Hoesel, & Schmid, 2013; Huminiecki et al., 2017; Israël, 2010). Our results further demonstrated that CUR treatment of EESCs completely inhibited or eliminated phosphorylated forms of JNK and STAT3, along with IKKα, IKKβ, and NF‐κB. Thus, our results are consistent with reports showing that CUR has strong antiproliferative, anti‐inflammatory, and antiangiogenic properties (Beevers, & Huang, 2011; Lee et al., 2013; Shen, & Ji, 2012). Moreover, CUR has no negative effects on serum cholesterol and lipoproteins and does not promote the development of a pseudopregnant endocrine state (Beevers, & Huang, 2011; Lee et al., 2013; Shen, & Ji, 2012).

Together, CUR’s anti‐inflammatory, antiproliferative, and proapoptotic effects may offer a well‐tolerated alternative to standard, approved antiendometriosis medications. Nonhormonal therapeutics of plant origin may be especially suitable for young women with severe symptoms of endometriosis‐associated pain who require a protracted duration of therapy (Figure 6). Additional experiments are underway to elucidate CUR’s precise anti‐inflammatory and potential fertility‐preserving effects in in vivo models of endometriosis. Our findings support the investigation of novel, natural, and safe therapeutics for future endometriosis treatment.

## CONFLICTS OF INTEREST

The authors declare that there are no conflicts of interest.

## AUTHOR CONTRIBUTIONS

I. C. and S. B. contributed to study concept and design, acquisition, analysis and interpretation of data, statistical analysis, and drafting of the manuscript. W. Z. and S. M. contributed experimental support. C. N. and N. S. contributed patient samples. W. E. T., R. N. T., N. S., and A. D. contributed analysis and interpretation of data and critical revision of the manuscript for important intellectual content.
